# A phase II randomized placebo-controlled double-blind study of salvage radiation therapy plus placebo versus SRT plus enzalutamide with high-risk PSA-recurrent prostate cancer after radical prostatectomy (SALV-ENZA)

**DOI:** 10.1186/s12885-019-5805-z

**Published:** 2019-06-13

**Authors:** Roche Kapoor, Matthew P. Deek, Riley McIntyre, Natasha Raman, Megan Kummerlowe, Iyah Chen, Matt Gaver, Hao Wang, Sam Denmeade, Tamara Lotan, Channing Paller, Mark Markowski, Michael Carducci, Mario Eisenberger, Tomasz M. Beer, Daniel Y. Song, Theodore L. DeWeese, Jason W. Hearn, Stephen Greco, Curtiland DeVille, Neil B. Desai, Elisabeth I. Heath, Stanley Liauw, Daniel E. Spratt, Arthur Y. Hung, Emmanuel S. Antonarakis, Phuoc T. Tran

**Affiliations:** 1 Department of Radiation Oncology and Molecular Radiation Sciences, Sidney Kimmel Comprehensive Cancer Center, Johns Hopkins Hospital, 1550 Orleans Street, CRB2 Rm 406, Baltimore, MD 21231 USA; 2 Department of Medical Oncology, Sidney Kimmel Comprehensive Cancer Center, Johns Hopkins Hospital, 1650 Orleans Street, CRB1 Rm 1M45, Baltimore, MD 21231 USA; 3 The James Buchanan Brady Urological Institute and Department of Urology, Johns Hopkins University School of Medicine, Baltimore, MD USA; 4 Department of Pathology, Johns Hopkins University School of Medicine, Baltimore, MD USA; 5 OHSU Knight Cancer Institute, Oregon Health & Science University, Portland, OR USA; 6 Department of Radiation Oncology, University of Michigan, Ann Arbor, MI USA; 7 Department of Radiation Oncology, University of Texas Southwestern Medical Center, Dallas, TX USA; 8 Karmanos Cancer Institute, Department of Oncology, Wayne State University School of Medicine, Detroit, MI USA; 9 Department of Radiation Oncology and Cellular Oncology, University of Chicago, Chicago, IL USA; 10 Department of Radiation Medicine, OHSU Knight Cancer Institute, Oregon Health & Science University, Portland, OR USA

**Keywords:** Recurrent prostate cancer, Salvage radiation therapy (SRT), High-risk prostate cancer, Enzalutamide, Prostatectomy

## Abstract

**Background:**

In men with a rising PSA following radical prostatectomy, salvage radiation therapy (SRT) offers a second chance for cure. Hormonal therapy can be combined with SRT in order to increase prostate tumor control, albeit with associated higher rates of treatment side effects. This trial studies the effectiveness of SRT combined with hormonal therapy using a more potent anti-androgen with a favorable side effect profile. Enzalutamide, a next generation selective androgen receptor antagonist, is approved by the Food and Drug Administration for the treatment of metastatic castrate-resistant prostate cancer (CRPC) where it has been shown to improve overall survival in combination with androgen deprivation therapy. The primary objective of this study is to evaluate the efficacy of combination SRT and enzalutamide for freedom-from-PSA-progression. Secondary objectives include time to local recurrence within the radiation field, metastasis-free survival and safety as determined by frequency and severity of adverse events.

**Methods/design:**

This is a randomized, double-blind, phase II, prospective, multicenter study in adult males with biochemically recurrent prostate cancer following radical prostatectomy. Following registration, enzalutamide 160 mg or placebo by mouth (PO) once daily will be administered for 6 months. Following two months of study drug, external beam radiotherapy to 66.6–70.2 Gray (Gy) will be administered to the prostate bed over 7–8 weeks while continuing daily placebo/enzalutamide. This is followed by two additional months of placebo/enzalutamide.

**Discussion:**

The SALV-ENZA trial is the first phase II placebo-controlled double-blinded randomized study to test SRT in combination with a next generation androgen receptor antagonist in men with high-risk recurrent prostate cancer after radical prostatectomy. The primary hypothesis of this study is that clinical outcomes will be improved by the addition of enzalutamide compared to standard-of-care SRT alone and pave the path for phase III evaluation of this combination.

**Trial registrations:**

ClinicaltTrials.gov Identifier: NCT02203695 Date of Registration: 06/16/2014. Date of First Participant Enrollment: 04/16/2015.

## Background

Prostate cancer is the second leading cause of cancer deaths in men. According to American Cancer Society estimates in 2018, as many as 164,690 American men will be diagnosed with prostate cancer, and nearly 29,430 will die of the disease [1]. The course of prostate cancer, from diagnosis to death, is best categorized as a series of clinical states. These clinical states involve the complex interplay of a network of signaling molecules that collectively promote net cell proliferation relative to cell death. Based on the extent of disease, hormonal status, and absence or presence of detectable metastases on an imaging study, the states are: localized disease, rising levels of prostate-specific antigen (PSA) after radiation therapy or surgery with no detectable metastases, and clinical metastases in the non-castrate or castrate state. Most men that ultimately die of prostate cancer die from metastatic castrate-resistant disease [2]. However, of the approximately 30,000 men that die of prostate cancer per year, the majority of these men originally presented with localized prostate carcinoma, which failed local therapy such as radical prostatectomy and progress. While a significant proportion of patients with clinically localized prostate cancer will be cured with definitive local therapy, those patients with high-risk features such as Gleason grade 8–10, positive lymph nodes, positive surgical margins or seminal vesicles invasion have a 50–75% chance of disease recurrence in 10 years [3–8]. Men who undergo prostatectomy and are found to have any of these features have cure rates of less than 25% after long-term follow-up [4, 9, 10]. Importantly, these men at the time of initial PSA relapse following surgery still represent potentially curable patients with salvage radiation [9].

The rate of post-prostatectomy salvage radiation therapy (SRT) has increased over the past 10 years in conjunction with increasing use of prostatectomy in men with high-risk localized prostate cancer. In these instances RT is an established standard of care salvage therapy in men with a persistently detectable post prostatectomy PSA or a delayed increase in PSA without evidence of metastatic disease on imaging [9, 11–18]. Given the lack of randomized trials evaluating SRT, its adoption as a standard therapy has mostly come from retrospective series, the largest of which was reported by Tendulkar et al., [9] which examined predictors of PSA control following SRT and demonstrated higher Gleason score, higher pre-SRT PSA, negative prostatectomy surgical margins, extra-prostatic extension, ADT use, SRT dose, and seminal vesicle involvement were associated with poor outcomes. Even with these improvements in identifying whom to select for salvage therapy, high quality evidence on SRT (e.g., RT technique, addition of androgen deprivation/androgen receptor blockade) is still absent [2]. Regardless, it is clear that SRT alone is not likely to afford high levels of Freedom from PSA progression (FFPP) or cure in high-risk individuals. In these individuals the addition of androgen deprivation therapy (ADT) can lower rates of biochemical failure, lower distant metastases and improve overall survival particularly in certain high-risk subsets of men with biochemical failure [19, 20]. Some controversy exists regarding the clinical benefit of hormonal therapy with SRT in more contemporary men who present with low pre-SRT PSA prior to initiation of SRT [2]. Regardless of whether the addition of ADT may increase the efficacy of SRT in modern patients, ADT is associated with the side-effects of hot flashes,, bone and muscle loss, fatigue, weight gain, sexual dysfunction and decreases in global quality of life often making it difficult for men to tolerate [2].

Enzalutamide is a second-generation androgen receptor signaling inhibitor with demonstrated activity in cells that overexpress the androgen receptor. Unlike previous androgen receptor blocker (ARB) agents, enzalutamide does not display any agonist properties and blocks translocation of the ligand-receptor complex into the nucleus preventing DNA binding [21–23]. Enzalutamide is currently approved for use in men with metastatic castrate-resistant prostate cancer where it has been shown to significantly prolong survival in patients who have progressed on ADT [21, 22, 24] and in this setting is superior to bicalutamide [25]. Enzalutamide is also generally well tolerated with improved quality of life metrics when compared to bicalutamide [25, 26]. There is also promising intermediate-term data on enzalutamide alone in men with biochemically recurrent prostate cancer following prostatectomy [27]. Furthermore, a recent preclinical study demonstrated potent enzalutamide radiosensitization of androgen-sensitive prostate cancer cells in vitro [28]. These properties make enzalutamide an ideal candidate to combine with SRT in biochemically recurrent prostate cancer with the hopes of improving outcomes while minimizing toxicity.

## Methods/design

This study was approved by the Research Ethics Boards of Johns Hopkins Hospital and all collaborating institutions. The SALV-ENZA Trial is registered at the US National Institutes of Health (ClinicalTrials.gov) #NCT02203695.

### Objectives

The primary efficacy endpoint is the rate of Freedom-from-PSA-progression (FFPP). FFPP is defined as the time from randomization to the date of PSA progression.

#### Secondary Objectives:

- Local recurrence within the radiation field (confirmed pathologically)
- Metastasis-free survival (MFS) rates. Metastasis-free survival will be defined as the time from the date of registration to date of evidence of systemic disease on bone scan or cross sectional imaging or time to death, which occurs first.
- Safety, feasibility, and tolerability as assessed by National Cancer Institute (NCI) Common Toxicity Scales (v4.0), quality of life (EPIC survey), and achievement of accrual goals.

#### Inclusion criteria

To be included in this study, adult male patients should meet all of the following criteria:

- Willing and able to provide written informed consent and Health Insurance Portability and Accountability Act (HIPAA) authorization for the release of personal health information.
- Males aged 18 years of age and above.
- Patients must have histologically confirmed adenocarcinoma of the prostate gland.
- Patients must have received primary treatment with radical prostatectomy.
- Patients must have evidence of biochemical (PSA) relapse after prostatectomy, defined by one rise in PSA above a baseline detectable value (≥0.05 ng/mL) using measurements taken at least 4 weeks apart from each other (all PSA values must be within 12 months of study entry).
- Patients must have pathological Gleason (pG) sum 8–10; or pG sum 7 and either pT3 or R1 disease (i.e. positive margins.).
- Patients must have an absolute PSA level between > 0.1 and < 0.7 ng/mL at the time of study entry.
- Patients must have non-metastatic (M0) disease, as defined by a lack of metastases seen on CT scan of the abdomen/pelvis and whole-body radionuclide ^99^Tc bone scan, (or sodium fluoride PET scan) taken within 3 months of study entry.
- Patients must have had node negative (pN0) disease found at the time of surgery. If a nodal dissection was not performed at the original surgery then patients must be N0, as defined by a lack of radiographic or clinical evidence of local-regional tumor recurrence, including pelvic lymph nodes ≥2 cm in short-axis diameter.
- Patients must have non-castrate levels of serum testosterone (≥ 150 ng/dL).
- Patients must not have previously received hormonal therapy (LHRH agonist, antiandrogen, or both), with the exception of neoadjuvant or adjuvant hormones given in conjunction with prostatectomy. In such cases, hormone therapy must have been administered for ≤6 months, discontinued ≥6 months ago, and serum testosterone must be ≥150 ng/dL.
- Patients must have ECOG performance status of 0–1, and life expectancy ≥3 years.
- Patients must have laboratory test results within the ranges listed below within 4 weeks of enrollment:
  - WBC ≥ 3000/mm^3^
  - Granulocytes ≥1500/mm^3^
  - Hemoglobin≥9 g/dL
  - Platelets ≥100,000/mm^3^
  - Bilirubin ≤1.8 mg/dL
  - ALT and AST ≤ 2.5 times the institutional upper limit of normal
  - Creatinine ≤1.8 mg/dL OR a calculated creatinine clearance ≥60 mL/hr.
- Patients must be disease-free from prior malignancies for ≥3 years, with the exception of non-melanoma skin cancers and superficial urothelial cancers.
- Patients must have the ability to swallow the study drug whole as a tablet or capsule.
- Throughout study, male patient and his female partner who is of childbearing potential must use 2 acceptable methods of birth control (1 of which must include a condom as a barrier method of contraception) starting at screening and continuing throughout the study period and for 3 months after final study drug administration or per local guidelines where these require additional description of contraceptive methods. Two acceptable methods of birth control thus include the following:
  - Condom (barrier method of contraception); AND
  - One of the following is required:
    - Established and ongoing use of oral, injected, or implanted hormonal method of contraception by the female partner.
    - Placement of an intrauterine device or intrauterine system by the female partner.
    - Additional barrier method: Occlusive cap (diaphragm or cervical/vault caps) with spermicidal foam/gel/film/cream/suppository by the female partner.
    - Tubal ligation in the female partner.
    - Vasectomy or other procedure resulting in infertility (e.g., bilateral orchiectomy), for > 6 months.
- Throughout the study, patients must use a condom if having sex with a pregnant woman.

#### Exclusion criteria

Patients that meet any of the criteria listed below will not be eligible for study entry:

- Currently active second malignancy
- Primary treatment with radiation therapy.
- Radiographic or clinical evidence of regional tumor nodal recurrence, including pathological pelvic lymph nodes ≥2 cm in short-axis diameter. Radiographic evidence of distant metastases is also an exclusion.
- Concurrent use of other antiandrogens, estrogen-like agents, or 5 alpha-reductase inhibitors.
- Use of systemic corticosteroids equivalent to prednisone 10 mg/day or higher at the time of study entry (inhaled corticosteroids are permitted).
- Concurrent use of other anti-cancer agents or treatments.
  Serious concurrent medical illnesses (including uncontrolled major cardiac, pulmonary, Child-Pugh C liver or psychiatric diseases) or active major infections (including HIV, Hepatitis A-C).
- Clinically significant cardiovascular disease including:
  - Myocardial infarction within 6 months of screening visit.
  - Uncontrolled angina within 3 months of screening visit.
  - Congestive heart failure New York Heart Association (NYHA) class 3 or 4, or subjects with history of congestive heart failure NYHA class 3 or 4 in the past, or history of anthracycline or anthracenedione (mitoxantrone) treatment, unless a screening echocardiogram or multi-gated acquisition scan (MUGA) performed within three months of the screening visit results in a left ventricular ejection fraction that is ≥45%.
  - History of clinically significant ventricular arrhythmias (e.g., ventricular tachycardia, ventricular fibrillation, torsade de pointes).
  - Prolonged corrected QT interval by the Fridericia correction formula (QTcF) on the screening electrocardiogram (ECG) > 470 msec.
  - History of Mobitz II second degree or third degree heart block without a permanent pacemaker in place.
  - Hypotension (systolic blood pressure < 86 mmHg or bradycardia with a heart rate of < 50 beats per minute on the Screening ECG, unless pharmaceutically induced and thus reversible (i.e. beta blockers).
  - Uncontrolled hypertension as indicated by a resting systolic blood pressure > 170 mmHg or diastolic blood pressure > 105 mmHg at the screening visit.
- Medications which lowers seizure threshold.
- History of seizure or any condition that may predispose to seizure including, but not limited to underlying brain injury, stroke, primary brain tumors, brain metastases, or alcoholism. Also, history of loss of consciousness or transient ischemic attack within 12 months of enrollment (Day 1 visit).
- Patients taking medications that may have adverse interactions with enzalutamide.

### Evaluation of randomization and blinding

**Fig. 1:**
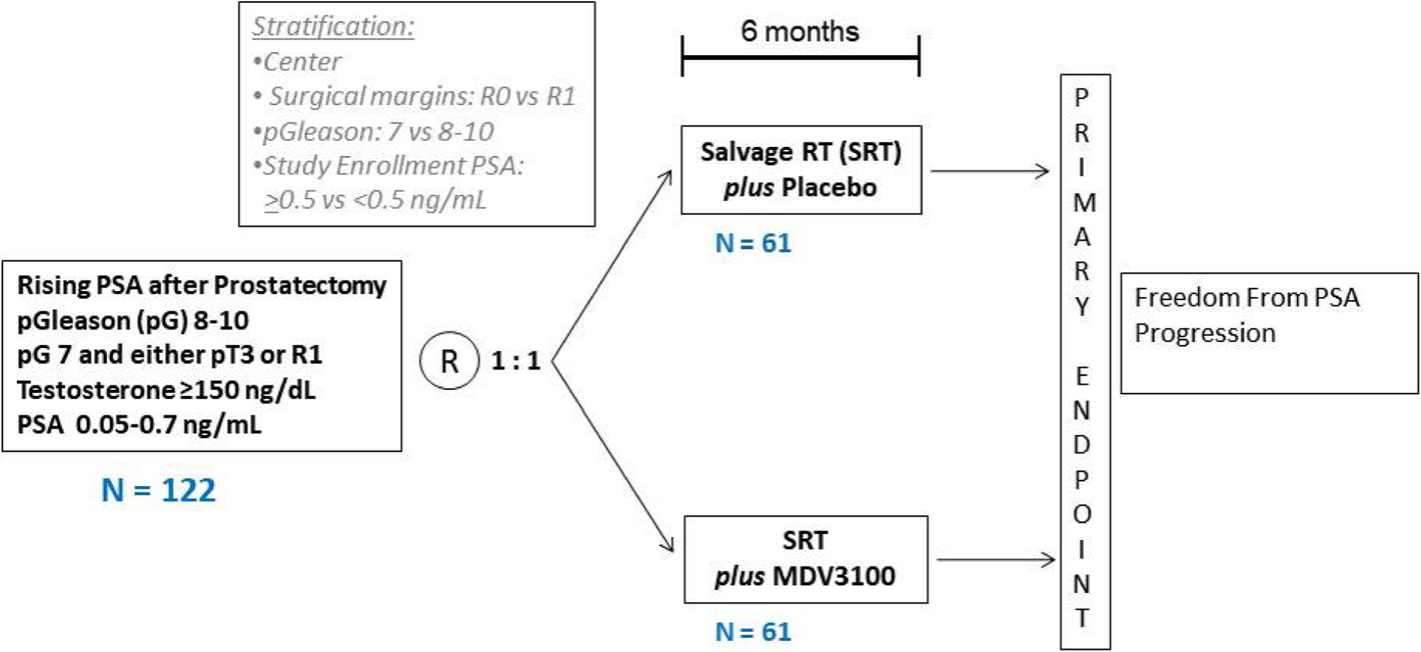
SALV-ENZA Study Schema. Subjects who meet eligibility criteria and qualify for enrollment will be stratified and randomized as demonstrated

This study is a multi-center, double-blind, placebo controlled, randomized Phase II trial in patients with non-castrate PSA-recurrent prostate cancer after radical prostatectomy. Patients were recruited from within the United States. Eligible patients will be randomized in a 1:1 ratio to one of two treatment arms: SRT plus placebo (Arm A) or SRT plus enzalutamide (Arm B) (Fig. 1). The study coordinator will use an interactive web response system (IWRS) will be utilized to obtain the patient’s randomization assignment. The randomization will be stratified by: center, surgical margin status (R0 vs R1), PSA prior to salvage treatment (PSA ≥0.5 vs < 0.5 ng/mL), and pathologic Gleason score (7 vs 8–10). Minimization approach (22) will be applied to ensure balanced assignment to each treatment arm. The investigator and the patient will be blinded to treatment assignment. The on-study date for protocol entry will be the day that the study subject is randomized. The principal investigator will be unblinded only if a patient progresses at any time during the study.

### Interventions

The screening/baseline procedures will determine patient eligibility according to the inclusion and exclusion criteria. The following evaluations/assessments will be performed at this visit *within 90 days* of Day 1:

- Obtain informed consent and research authorization.
- Obtain histologic and radiologic confirmation of disease.
- Collect details and dates of the primary therapy (e.g., pathologic stage, dose and type of radiation therapy) and prior hormonal and non-hormonal therapies.
- Record PSA and Gleason score at the time of diagnosis
- Determine suitability for salvage prostate bed radiation therapy
- Assess presence or absence of disease in the primary site
- Imaging
  - Chest by plain radiograph or computerized tomography (CT)
  - Abdomen/pelvis by CT or magnetic resonance imaging (MRI)
  - Radionuclide bone scan

The following assessments must occur within 30 days of registration/randomization:

- Physical exam (vital signs, height/weight, ECG, etc.)
- Laboratory tests (CBC w/diff, PSA, testosterone, comprehensive chemistry panel, including bilirubin, creatinine, SGOT[AST], SGPT[ALT])
- ECOG performance status
- Review of concurrent medications

The following procedures are to be conducted each study visit on visit on Day 1, 61, 91, 120, 151 and 180 days while on study. Day 1, 61, 120 procedures should be done within seven days prior; day 91,151 and 180 procedures should be done +/− 14 days:

- Review concurrent medications
- Physical exam (vital signs, weight)
- ECOG performance status
- Adverse events evaluation
- Review pill diary
- Laboratory tests (CBC w/diff, PSA, testosterone, comprehensive chemistry panel, including bilirubin, creatinine, SGOT[AST], SGPT[ALT]
- Quality of Life (QoL) questionnaires
- Decipher Test (Will only be completed once before treatment ends on Day 180. This test will not be repeated.)

The following procedures are to be conducted at each follow-up visit every 3 months ±1 month up to 24 months:

- Review concurrent medications
- Physical exam (vital signs, weight)
- ECOG performance status
- Adverse events evaluation
- Laboratory tests (CBC w/diff, PSA, testosterone, comprehensive chemistry panel, including bilirubin, creatinine, SGOT[AST], SGPT[ALT]
- QoL questionnaires

The following procedure is to be conducted at each follow-up visit every 3 months ±1 month past the first 24 months and up to 42 months:

- Laboratory test (PSA)

Patients will be followed for > 2 years (and up to 42 months total) after removal from treatment or until death. Patients withdrawn from the study because of adverse events (AE) will be followed until the adverse event has either resolved or stabilized. Reasons for premature withdrawal should be determined and noted.

AEs will be monitored at each scheduled visit and throughout the study. Toxicity will be assessed using the most recent NCI guidance: the most recent version of Common Terminology Criteria for Adverse Events (CTCAE). All nonserious AEs and serious adverse events, regardless of relationship to study treatment, will be collected from registration through the post-treatment visit and will be recorded in the subject’s medical record and on the case report form (CRF). Following the post-treatment visit, only new treatment-related AEs and SAEs will be recorded. Seizures (regardless of causality) will be recorded throughout the study.

### Radiation planning and dosage

Radiation planning and dosage are based off of post-operative salvage radiation consensus guidelines and RTOG 0534 [29].

A treatment planning CT scan will be used to define the clinical and planning target volumes, and the critical normal structures. The treatment planning CT will be acquired with the patient supine, set up in the same position as for daily treatments, and immobilized using a combi-fix. The CT scan of the pelvis should start at or above the iliac crest down to below the perineum (below the ischial tuberosities). All tissues to be irradiated must be included in the CT scan. CT scan thickness should be ≤0.5 cm through the region that contains the target volumes (i.e., from the bottom of the sacroiliac joints down to the penile urethra). The regions above and below the target volume region may be scanned with slice thickness ≤ 1.0 cm. Contrast may be used for simulation but can distort the anatomy slightly and so is not recommended. The bladder should be reasonably full for simulation, keeping in mind that patients may not be able to maintain as full a bladder during radiotherapy. Having a full bladder at simulation ensures that the clinical target volume (CTV) will be of maximal dimensions.

#### Clinical target volume (CTV) (prostate bed)

The CTV will extend from the top of the penile bulb inferiorly, or 1.5 cm below the urethrogram peak if done, to just above the pubic symphysis superiorly (at least for the anterior-most portion of the bladder). Laterally, the CTV will extend from the medial edge of one obturator internus muscle to the other. Anteriorly the CTV will include the entire bladder neck until the mid-pubic symphysis, where a gradual reduction off of the anterior bladder is made. Posteriorly, the CTV is defined by the anterior-most aspects of the anus-rectum. The CTV may be increased (not decreased) beyond these limits based on pre-prostatectomy imaging information. The seminal vesicles or remnants thereof, if identified on CT or MRI as being present, will receive the full dose. The immediate periprostatic bed surgical clips should receive the full dose. The pelvic lymph nodes are not to be included in the CTV on this protocol.

#### Planning target volume (PTV)

The PTV margins should be a 0.5–1.5 cm in all dimensions. 95% of the PTV must receive the prescribed dose. Care should be taken to conform the prescribed dose as closely to the PTV as possible, so as to avoid including the entire width of the rectum in the posterior blocked margin at the bladder neck-rectum interface. The maximum dose heterogeneity allowable in the PTV will be 10%; a variation will be > 10% and a violation > 15%.

The critical normal structures are the bladder, rectum, and femoral heads. The normal tissues will be contoured and considered as solid organs.

- The bladder should be contoured from its base to the dome, excluding the CTV (the CTV includes the bladder neck).
- The rectum should be contoured from the anus (at the level of the ischial tuberosities) to the rectosigmoid flexure (this is roughly at about 10 cm) or for a maximum length of 15 cm if the sigmoid flexure if felt to be higher.
- Each femoral head should be outlined down to the interface between the greater and lesser trochanters.

### Early stopping guidelines

In the absence of treatment delays because of adverse events, treatment will continue for 6 months or until one of the following criteria applies:

- Patient decides to withdraw from the study
- Disease progression
- Symptomatic disease progression at any time
- Objective clinical disease progression
- Intercurrent illness that prevents further administration of treatment
- Unacceptable adverse event(s) that may or may not be directly related to treatment but that, in the judgment of the treating physician, makes it dangerous for the patient to be retreated
- General or specific changes in the patient’s condition that render the patient unacceptable for further treatment, in the judgment of the investigator

Because an excessive rate of withdrawals can render the study uninterpretable, unnecessary withdrawal of patients should be avoided. When a patient discontinues treatment early, the investigator should make every effort to contact the patient and to perform a final evaluation. The reason(s) for withdrawal should be recorded.

### Statistical analysis

#### Sample size and accrual

Based on the largest multi-institutional SRT series published at the time of this study design in which patients received SRT followed by observation until the PSA reached ≥0.2 ng/mL above the post-SRT nadir, the 2-year FFPP was approximately 60% with SRT [30]. For our primary endpoint of 2-year FFPP we expect an absolute 20% improvement with the treatment of SRT+ enzalutamide (2-year FFPP 80%) over SRT alone (2-year FFPP 60%).

We assume an accrual time of 18 months, with > 24 months of additional follow-up time. FFPP at 2 years improved from 60 to 80%, a 20% increase (corresponding to a hazard ratio of 0.44 under the assumption of exponential distribution of event times) corresponds to 39 events total of PSA progression (for example 26 in Arm A, and 13 in Arm B) with a 90% power to detect an improvement of, using a one-sided log-rank test at significance level 0.1. Adjusting for 15% non-evaluable or dropout patients, we will randomize a total of 122 patients (61 patients in each arm).

The power of the study is driven by the number of PFS progression events. The design requires 39 events when the analysis of the primary endpoint is conducted. Under the assumption that there are not non-evaluable patients or no dropout, a total of 102 patients need to be enrolled over 18 months with > 24 months of additional follow-up to reach 39 events. Because the study duration is as long as 3.5 years, some patients may be lost to follow-up before that and their time to PSA progression will be censored at the last date of PSA measurement. A non-evaluable and loss-to-follow-up rate of 15% is used to estimate the total number of participants.

#### Data analysis

- FFPP will be summarized using Kaplan-Meier method by treatment arms. FFPP curves will also be displayed graphically. Differences in FFPP between treatment arms will be compared by the log rank test. The Cox proportional hazards model will be fitted, and the estimated hazard ratio (Arm B/Arm A) and corresponding 95% CI will be provided.
- FFPP probability will be estimated using the Kaplan-Meier method
- Additionally, Cox regression models will be used to explore the potential influences of the other factors on the primary FFPP endpoints.
- Time-to-event endpoints, time to local recurrence, and radiographic MFS will be analyzed similarly as described for the FFPP.
- For safety analysis, overall safety profile and toleration of Arm A and Arm B will be characterized by type, frequency, severity, timing and relationship of study therapy of adverse events and laboratory abnormalities. Adverse events will be summarized by the frequency of patients experiencing treatment emergent adverse events corresponding to body systems and by worst NCI CTCAE (version 4.0) grade.

### Response criteria

#### Analysis of the primary endpoint

The primary objective of the study is to evaluate the efficacy of the two treatment regimens based on freedom-from-PSA-progression (FFPP). FFPP is defined as the time from randomization to the date of PSA progression. A subject who does not have PSA progression at the time of the analysis will be censored at the last date of PSA measurement. In patients who achieve an undetectable PSA value (defined as ≤0.1 ng/mL), PSA progression is defined as a detectable PSA value (≥0.2 ng/mL) that is confirmed by a second consecutive PSA value obtained ≥8 weeks later which is higher (and ≥ 0.4 ng/mL). In patients who do not achieve an undetectable PSA, PSA progression is defined as a 0.2 ng/mL increase from nadir that is confirmed by a second consecutive PSA value obtained ≥8 weeks later which is higher.

It is very unlikely that deaths in the time frame of this trial will be from prostate cancer. Censoring the subjects that have died at the date of the last PSA measurement before death will be performed (as we will not know if PSA changes in the interval) unless there is clear evidence of death from prostate cancer or treatment per the treating physician and/or by autopsy.

#### Analysis of secondary endpoints

The first secondary endpoint is time to local recurrence within the radiation field, defined as the time from randomization to the date of local recurrence of disease. Subjects who do not have local recurrence will be censored on the date of their last evaluable tumor assessment for local recurrence. Subjects who start any subsequent anti-cancer therapy without a prior reported local recurrence will be censored at the last evaluable tumor assessment prior to initiation of the subsequent anti-cancer therapy.

The second secondary endpoint is MFS, defined as the time from randomization to the date of metastasis or death from any cause up to 90 days following the last response assessment, whichever occurs first. A subject who does not have metastasis and is alive will be censored at the last date of tumor assessment.

Time-to-event endpoints (time to local recurrence and MFS) will be analyzed similarly as described for the primary endpoint (FFPP).

Adverse events will be summarized by the frequency of patients experiencing treatment emergent adverse events corresponding to body systems and by worst NCI CTCAE (version 4.0) grade.

## Discussion

Certain high-risk features, such as Gleason score 8–10, positive lymph nodes, positive surgical margins or seminal vesicles invasion, will put men with prostate cancer at high risk for disease recurrence following radical prostatectomy [3–8]. In these individuals with biochemically recurrent disease, SRT offers a second chance for cure. However, despite this, rates of failure after SRT remain high [9]. The addition of ADT to this regimen may improve its clinical effectiveness and decreases the risk of biochemical failure, but does so at the cost of increased side effects. The SALV-ENZA trial builds on the prior RTOG 96–01 and GETUG-16 studies, while using a newer more potent anti-androgen enzalutamide. Whether the addition of hormonal therapy to SRT improves distant metastases and survival in a more contemporary high-risk cohort of men who present with low pre-SRT PSA is an area in need of investigation [2]. SALV-ENZA is investigating the safety and effectiveness of adding enzalutamide to SRT for just such a high-risk cohort of men who have rising PSA levels following prostatectomy. SALV-ENZA is among the first phase II placebo-controlled double-blinded randomized studies to test SRT in combination with a next generation anti-androgen receptor antagonist in men with contemporary high-risk recurrent prostate cancer after radical prostatectomy. However, a number of additional SRT phase II randomized studies investigating other next generation anti-androgen receptor antagonists are now activated or soon will be (NCT03141671 and NCT03371719). We hope together these collective trials illuminate possibly better treatment options for these high-risk men with biochemical failure following prostatectomy and facilitate further interrogation in the phase III setting.

## Data Availability

Raw data is not currently available for publication as the trial is still accruing patients and has not undergone interim analysis.

## Abbreviations

AE: Adverse event
ALT: Alanine aminotransferase
ARB: Androgen receptor blocker
AST: Aspartate aminotransferase
CRF: Case report form
CRPC: Castrate-resistant prostate cancer
CT: Computerized tomography
CTCAE: Common terminology criteria for adverse events
CTV: Clinical target volume
ECG: Electrocardiogram
FDA: Food and Drug Administration
FFPP: Freedom from PSA progression
Gy: Gray (unit)
HIPAA: Health Insurance Portability and Accountability Act
HIV: Human immunodeficiency virus
IRB: Institutional Review Board
IWRS: Interactive web response system
LHRH: Luteinizing hormone-releasing hormone
M0: Non-metastatic
MFS: Metastasis-free survival
MRI: Magnetic resonance imaging
MUGA: Multi-gated acquisition scan
NCI: National Cancer Institute
NYHA: New York Heart Association
pG: Pathological Gleason
pN0: Node negative
PO: Per os (by mouth)
PSA: Prostate-specific antigen
PSADT: Prostate-specific antigen doubling time
PTV: Planning target volume
QoL: Quality of life
SAE: Serious adverse event
SGOT: Serum glutamic oxaloacetic transaminase
SGPT: Serum glutamic-pyruvic transaminase
SKCCC: Sidney Kimmel Comprehensive Cancer Center
SRT: Salvage radiotherapy
WBC: White blood cell
